# B-Cell Epitope Mapping of the *Treponema pallidum* Tp0435 Immunodominant Lipoprotein for Peptide-Based Syphilis Diagnostics

**DOI:** 10.3390/diagnostics15111443

**Published:** 2025-06-05

**Authors:** Jessica L. Keane, Mahashweta Bose, Barbara J. Molini, Kelika A. Konda, Silver K. Vargas, Michael Reyes Diaz, Carlos F. Caceres, Jeffrey D. Klausner, Rebecca S. Treger, Lorenzo Giacani

**Affiliations:** 1 Department of Medicine, Division of Allergy and Infectious Diseases, University of Washington, Harborview Medical Center, 300 9th Ave, Seattle, WA 98104, USA; jessica.keane@yale.edu (J.L.K.); mahashweta.c.bose@gmail.com (M.B.); bjmolini@uw.edu (B.J.M.); 2 Keck School of Medicine, University of Southern California, 1975 Zonal Ave, Los Angeles, CA 90033, USA; kelikakonda@gmail.com (K.A.K.); jklausne@usc.edu (J.D.K.); 3 Center for Interdisciplinary Studies in Sexuality, AIDS and Society, Universidad Peruana Cayetano Heredia, Av. Honorio Delgado 430, Lima 15102, Peru; silver.vargas.r@upch.pe (S.K.V.); edward.reyes@upch.pe (M.R.D.); carlos.caceres@upch.pe (C.F.C.); 4 Department of Laboratory Medicine and Pathology, University of Washington, 1959 NE Pacific St., Seattle, WA 98195, USA; rstreger@uw.edu; 5 Department of Global Health, University of Washington, Harborview Medical Center, 300 9th Ave, Seattle, WA 98104, USA

**Keywords:** syphilis, epitope mapping, *Treponema pallidum*, Tp0435 lipoprotein

## Abstract

**Background/Objectives:** Syphilis, a chronic sexually transmitted disease caused by the spirochete *Treponema pallidum* subspecies *pallidum* (*T. pallidum*), is still endemic in low- and middle-income countries and has been resurgent for decades in many high-income nations despite being treatable. Improving our understanding of syphilis pathogenesis, immunology, and *T. pallidum* biology could result in novel measures to curtail syphilis spread, including new therapeutics, a preventive vaccine, and, most importantly, improved diagnostics. **Methods:** Using overlapping synthetic peptides spanning the length of the *T. pallidum* Tp0435 mature lipoprotein, an abundant antigen known to induce an immunodominant humoral response during both natural and experimental infection, we evaluated which Tp0435 linear epitopes are most significantly recognized by antibodies from an infected host. Specifically, we used sera from 63 patients with syphilis at different stages, sera from non-syphilis patients (*n* = 40), and sera longitudinally collected from 10 rabbits infected with either the Nichols or SS14 isolates of *T. pallidum*, which represent the model strains for the two known circulating clades of this pathogen, to further evaluate the use of this animal model for syphilis studies. Recognized amino acid sequences were then mapped to the experimentally determined Tp0435 structure. **Results:** Reactive epitopes in both serum groups mapped predominantly to the α-helix preceding Tp0435 soluble β-barrel and the loops of the barrel. **Conclusions:** In the current effort to improve current syphilis diagnostics, the peptides corresponding to these immunodominant epitopes could help develop epitope-based assays such as peptide-based ELISAs and lateral flow point-of-care tests to improve the performance of treponemal tests and expedite diagnosis in low-income settings, where the infection is still a significant concern for public health and access to facilities with laboratories equipped to perform complex procedures might be challenging.

## 1. Introduction

Syphilis is a chronic sexually transmitted infection caused by the spirochete bacterium *Treponema pallidum* subsp. *pallidum* (*T. pallidum*) that, despite being treatable, is still a significant concern for local and global health. If the infection is left untreated due to the lack of a timely diagnosis, or it is not properly treated because of (a) the use of a now virtually ineffective antimicrobial such as azithromycin, (b) a therapeutic regimen not appropriate for the stage of the disease, or (c) incomplete adherence to proper treatment, syphilis can progress to affect the cardiovascular and central nervous systems, possibly leading to early and late neurosyphilis, aortic aneurysm, stroke-like syndrome, and death [1].

The WHO estimates an annual global incidence of 8 million new cases among individuals aged 15–49 in 2022, compared to 7.1 million in 2020 [2]. Although most of these cases occur in low- and middle-income countries (LMICs), syphilis has been resurgent over the last two decades in several high-income nations of North America, Europe, Asia, and Australia [3,4], particularly in men who have sex with men (MSM), minorities, and persons living with HIV (PLHIV) [5,6]. Although the most recent data for the year 2023 from the CDC show that, in the U.S., this resurgence might be slowing down, with only a 1% increase in overall syphilis cases compared to 2022 [5], epidemiological data are still concerning when compared to the all-time low levels of syphilis recorded in 1999, when syphilis elimination in the U.S. seemed within reach.

Congenital syphilis, caused by transplacental transmission of the pathogen, has undergone an even more dramatic increase, with 1.5 million cases worldwide in 2023 as compared to 700,000 in 2016 [7]. It therefore continues to significantly impact the health of women of reproductive age and neonates in LMICs [8].

The current epidemiology of syphilis supports the need for research endeavors to deliver novel and effective control measures to curtail syphilis spread. Such endeavors range from developing novel diagnostic tools or improving existing ones, repurposing drugs approved for other infections but known to be effective against *T. pallidum* [9,10], to identifying effective syphilis vaccine formulations that can move into clinical trials.

Syphilis diagnosis is not always straightforward. With some exceptions [11], primary chancres are painless and may occur on the patient’s cervix or rectum, making them easy to go unnoticed during routine exams. Because secondary manifestations can be misleading due to the protean symptoms of syphilis, the combination of patient anamnesis and serologic tests is the most common way syphilis diagnosis is achieved [12]. During latency, when no clinical manifestations are present to inform diagnosis, serological tests are the only available instrument. Although direct pathogen detection via nucleic acid amplification tests (NAATs) has been shown to be helpful in complementing serology and molecular tests to detect *T. pallidum* in biological specimens are available in CLIA-certified laboratories whether or not they are FDA approved [13,14,15], these tests are affected by the pathogen clearance process preceding the latent stages that occurs as specific immunity to the pathogen develops, and although a positive pre-treatment molecular test result is indicative of an active infection, a negative result cannot rule it out entirely.

Syphilis serological tests are divided into lipoidal tests (LTs, also referred to as non-treponemal tests, now considered a misnomer) and treponemal tests (TTs). Lipoidal tests, such as the rapid plasma reagin (RPR) test, measure antibodies (IgM or IgG) to a cardiolipin/cholesterol/lecithin antigen from damaged host cells and bacterial membranes during infection [16,17,18]. Without treatment, LT antibody titers peak one to two years after infection and remain positive even in late stages of the disease [19,20]. In immunocompetent patients, titers decline after treatment and may become nonreactive within six months, even though, in some cases, up to two years might be needed [21,22]. A four-fold decrease in LT titers is associated with serological cure, while reinfection generally causes a titer increase. Lipoidal tests are, therefore, instrumental in diagnosing active syphilis cases and assessing response to therapy [19,20,23]. Treponemal tests such as the *T. pallidum* particle agglutination (TPPA) and *T. pallidum* hemagglutination (TPHA) tests use whole *T. pallidum* cell extracts to detect antibodies to treponemal antigens [23]. In recent years, however, treponemal enzyme and chemiluminescence immunoassays (EIA and CIA, respectively) employing immunodominant recombinant antigens such as Tp0171 (TpN15), Tp0435 (TpN17), and Tp0574 (TpN47) have come into widespread clinical use, as these assays can be performed using high-throughput automated instruments [24]. Because treponemal antibodies persist for the lifetime of the patient, TTs cannot discriminate between active and previously treated infections. These tests become positive one to two weeks after the chancre appears and may be helpful to detect early cases before LT titers rise [23].

Due to their chemical versatility, durability, and accessibility compared to full-length proteins, peptides and peptidomimetics have generated interest in their potential to be used in diagnostic tests. Synthetic peptides that effectively recapitulate the serological recognition of the larger proteins from which they are derived could eventually replace recombinant antigens, which would facilitate test manufacturing and improve their affordability. Here, we report a study conducted to gain knowledge of the sequences of the Tp0435 immunodominant lipoprotein that are naturally immunogenic during infection. We selected this protein because, in addition to being an immunodominant antigen and highly conserved in *T. pallidum* strains sequenced so far [25], its crystal structure has been reliably determined at a resolution of 2.42 Å [26]. The structure of this protein is characterized by an eight-stranded β-barrel protein with a basin at one end of the barrel and an α-helix stacked on the opposite end, implying plausible roles for the protein in either ligand binding or treponemal membrane architecture. To fulfill our goal, we performed B-cell epitope mapping of this antigen using linear, overlapping synthetic peptides spanning the entirety of the mature Tp0435 (i.e., lacking the cleavable signal peptide), as well as sera collected from 63 patients with syphilis at different stages and 40 sera from patients with negative serology for syphilis, which we equated to “healthy controls” (see Institutional Review Board Statement).

Furthermore, we also evaluated reactivity to these peptides of sera longitudinally collected from two groups of five rabbits each, one infected with the Nichols strain of *T. pallidum* and the second with the SS14 strain, which are the two laboratory strains representing the two clades of *T. pallidum* strains circulating worldwide [25].

## 2. Materials and Methods

### 2.1. Ethics Statement

Male New Zealand White (NZW) rabbits (*Oryctolagus cuniculus*) ranging from 3.5–4.5 kg in weight were used in this study. Specific pathogen-free (SPF; *Pasteurella multocida* and *Treponema paraluiscuniculi*) animals were purchased from Western Oregon Rabbit Company (Philomath, OR, USA) and housed at the University of Washington (UW) Animal Research and Care Facility (ARCF). Care was provided according to the procedures described in the Guide for the Care and Use of Laboratory Animals [27] under protocols approved by the UW Institutional Animal Care and Use Committee (IACUC; Protocol # 4243-01, PI: Lorenzo Giacani). Upon arrival and before enrollment in the study, all rabbits were bled, and sera were tested with a treponemal (TPPA; Fujirebio, Tokyo, Japan) and a lipodal test (VDRL; Becton Dickinson, Franklin Lakes, NJ, USA) to confirm lack of immunity to *Treponema paraluiscuniculi*, given that animals are tested randomly by the provider. Both tests were performed according to the manufacturer’s instructions. Only seronegative rabbits were used for experimental infection with the Nichols or the SS14 strains of *T. pallidum*. The Animal Research: Reporting of In Vivo Experiments (ARRIVE) guidelines were followed whenever applicable for experimental design involving animals and reporting of results.

### 2.2. Study Sites and Population

The human sera used in this study represent a subset of samples from patients recruited between 2019 and 2021 from five sexual health clinics, three of which are located in metropolitan areas of Lima (Independencia, San Juan de Lurigancho, and Barranco districts, respectively), one in the Callao region, and one in Eastern Peru in the city of Pucallpa, in the context of the PICASSO study [28]. All centers provide testing and care for STIs. To be eligible for enrollment, participants had to be 18 or older and newly diagnosed with active syphilis, with documentation of prior syphilis testing and/or treatment at the recruitment site and willingness to provide study-related biological specimens. Patients were asked to return to the site for follow-up visits at 3 and 6 months post-treatment for evaluation of treatment success. Individuals recruited in this study could either be at their first syphilis infection or have a history of infection. Participants were defined as syphilis naïve if they had a newly reactive RPR at enrollment, a positive TPPA test, and a documented negative treponemal antibody test taken within the prior 12 months. Repeat syphilis infection was defined by a new four-fold titer increase or a newly reactive RPR with a previously RPR test titer ≥ 1:8 or a previously documented positive treponemal antibody test. Disease staging into primary, secondary, early latent (when the infection likely occurred within the last 12 months), or late latent stage (when the infection likely occurred more than one year prior to the visit) was performed at recruitment according to the US CDC guidelines available at https://ndc.services.cdc.gov/case-definitions/syphilis-2018/ (Accessed on 4 April 2025).

Subjects meeting eligibility criteria and willing to participate signed an informed consent form and received appropriate reimbursement for transportation costs. Overall, a total of 63 patient samples were used in this study: 22 were diagnosed with primary syphilis, 7 with secondary syphilis, 22 with early latent infection, and 12 with late latent infection. Cohort data are reported in Table 1.

### 2.3. Rabbit Infection and Sera Collection

Ten rabbits were randomly assigned to two groups of five animals each and were infected intradermally (ID) with either the Nichols or SS14 *T. pallidum* strain at six sites on their shaved backs. Each site received 10^6^ treponemes, for a total of 6 × 10^6^ pathogen cells. Treponemal cells for ID inoculation were harvested the day of ID infection from donor rabbits previously infected intratesticularly (IT) to ensure that enough *T. pallidum* cells would be available for ID inoculation. The procedures for in vivo propagation of *T. pallidum* via IT infection were described in detail by Lukehart and Marra [29]. Treponemes were harvested from IT-infected animals at peak orchitis, and motility was checked pre- and post-ID inoculation to ensure the viability of virtually all inoculated treponemal cells. Serum samples were collected from the ID-infected rabbits at day 40 and day 90 post-infection and used for epitope mapping as described below. Pre-infection sera were collected for background measurement.

### 2.4. Recombinant Tp0435 Expression and Purification

Tp0435 protein was expressed as a full-length mature protein (i.e., lacking the predicted cleavable signal peptide, identified using PrediSi (http://www.predisi.de/, accessed on 20 March 2025) and/or SignalP 5.0 (https://services.healthtech.dtu.dk/service.php?SignalP-5.0, accessed on 20 March 2025). The coding sequence was amplified from the Nichols strain genome of *T. pallidum* [30] (NC_021490.2/CP004010.2), and was cloned into the pEXP-5-NT (Life Technologies, Carlsbad, CA, USA) plasmid, which adds an amino-terminal His-tag. Post-cloning sequence accuracy was assessed by Sanger sequencing. The plasmid was then transformed into *E. coli* Rosetta2 DE3 pLysS BL21 cells (Millipore Sigma, Burlington, MA, USA), and cells were grown in auto-inducing media prepared according to Studier et al. [31] at room temperature for 36 h. Protein expression and solubility prior to purification were assessed by performing sodium dodecyl sulfate polyacrylamide gel electrophoresis (SDS-PAGE) separation and western blot with anti-His antibodies (Millipore Sigma, Burlington, MA, USA) as previously described [32]. Recombinant Tp0435 was purified using a nickel-based affinity chromatography system (Qiagen, Germantown, MD, USA) under non-denaturing conditions directly from the culture supernatant after cell sonication and centrifugation/filtering of the lysate to eliminate cellular debris. Protein was dialyzed against 1X phosphate-buffered saline (PBS). Protein size and purity were assessed using SDS-PAGE, and concentration was determined via a bicinchoninic acid assay kit (Fisher Scientific, Hampton, NH, USA).

### 2.5. ELISAs with Overlapping Synthetic Peptides and Recombinant Tp0435

To perform epitope mapping of the Tp0435 antigen, 24 linear synthetic 20-mer peptides, each overlapping by 15 amino acids, were designed to span the mature protein sequence (aa 25–159) of the Nichols and SS14 strains of *T. pallidum* devoid of its signal peptide, encompassing aa 1–24. Peptides were manufactured by GenScript (Piscataway, NJ, USA). Lyophilized peptides were reconstituted per manufacturer’s instructions with sterile 1X PBS to a final concentration of 1 mg/mL and stored at −20 °C until use. The solubility of slightly hydrophobic peptides (peptides # 2, 6, 8, 14, and 15) was increased by adding up to 4% (*v*/*v*) DMSO per the manufacturer’s instructions when needed. Peptide sequences are reported in Table 2.

The ELISA procedure was performed as already reported [34]. Briefly, peptides were further diluted to 10 μg/mL in 1x PBS, and 50 μL of working dilution (500 ng total) was used to coat the wells of a 96-well microwell Maxisorp flat-bottom immunoplate (Thermo-Fisher, Waltham, MA, USA). Control wells were also coated with recombinant Tp0435 protein. Coated plates were incubated at 37 °C for 2 h and then at 4 °C overnight to allow peptides to adhere. Wells were then blocked with 5% non-fat milk in 1x PBS (NFM-PBS) on a shaking platform at room temperature for 2–3 h. Sera diluted 1:100 in 1% NFM-PBS were added, and plates were incubated overnight at 4 °C. Either a secondary goat anti-human or a goat anti-rabbit IgG antibody conjugated with alkaline phosphatase (both from Millipore-Sigma, St. Louis, MO, USA) was used to develop plate wells. Secondary antibodies were used at a 1:2000 dilution. Plates were developed with 50 μL/well of 1 mg/mL para-nitrophenylphosphate substrate (pNPP; Millipore-Sigma) for 1 h, and absorbance was measured at 405 nm using a Synergy HTX multi-mode plate reader with the 3.10.6 version of the Gen5 Microplate Reader and Imager Software (BioTek, Winooski, VT, USA). Each serum sample was tested in triplicate. The mean ± SE of triplicate experimental wells, minus the mean of the wells with peptides tested with either pre-infection sera (in the case of rabbit sera) or sera from non-syphilis patients, was calculated. The resulting values were graphed using Prism software (Version 9.5.1; GraphPad, San Diego, CA, USA). For the graphic representation of reactivity, the PyMOL Software (https://www.pymol.org/; version 2.5; accessed on 5 February 2025) was used to visualize the structure of the Tp0435 protein (PDB ID: 4U3Q).

## 3. Results

### 3.1. Reactivity of Patient Sera to Tp0435 Peptides

**Figure 1:**
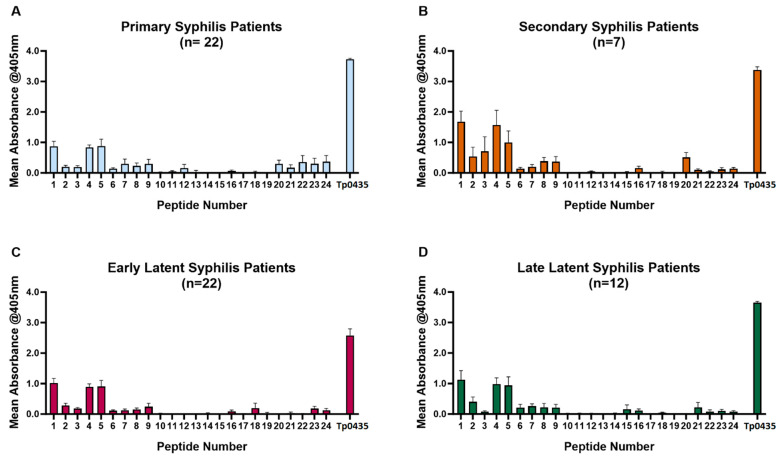
Reactivity of sera from syphilis patients to Tp0435 peptides. Mean reactivity to Tp0435 peptides in sera collected from primary (**A**), secondary (**B**), early latent (**C**), and late latent (**D**) syphilis sera. In all graphs, the Tp0435 label represents reactivity to wells where the full-length Tp0435 antigen was plated.

**Figure 2:**
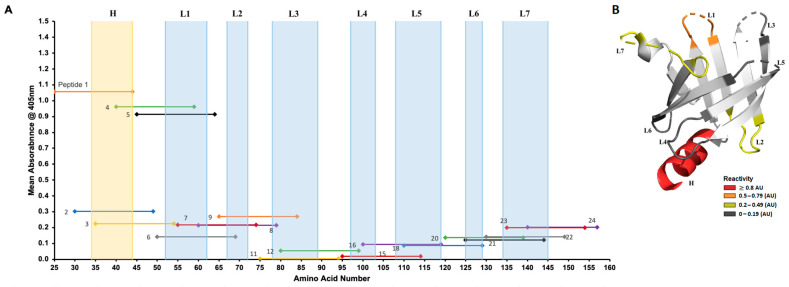
Mean reactivity of patient sera to individual peptides mapped to the Tp0435 protein structure. (**A**) Mean reactivity to individual peptides in patient sera independently of disease stage. The *x* axis represents individual Tp0435 amino acids (25–159) without the cleavable signal peptide. Amino acids corresponding to the Tp0435 amino-terminal helix (H) and loops (L1–L7) are represented in the background highlighted in blue. (**B**) Tp0435 loops and amino-terminal sequence are highlighted in the experimentally determined protein structure based on mean reactivity in human sera. Loops with a mean reactivity between ≤0.19 absorbance units (AUs) are highlighted in dark gray. Loops with a mean reactivity between 0.2 and 0.49 AUs are yellow, loops with a mean reactivity between 0.5 and 0.79 AUs are in orange, and loops with a mean reactivity ≥ 0.8 AUs are in red.

The analysis of sera from patients with primary (*n* = 22), secondary (*n* = 7), early latent (*n* = 22), and late latent (*n* = 12) syphilis to Tp0435-derived peptides (Figure 1A–D) showed that, regardless of the disease stage, reactivity was mainly directed to peptides 1–9 mapping to the amino-terminal region of Tp0435 (Table 2; aa 25–84). Within this region, however, sera were most reactive to peptides 1, 4, and 5 (Figure 1A–D), while peptides 2, 3, and 6–9,were recognized to a lesser extent. Patient sera were also slightly reactive to peptides 20–24, corresponding to the carboxyl terminus (Table 2; aa 120–159) of the antigen. Reactivity to peptides 20–24 was mainly seen in early (primary and secondary) syphilis samples rather than in late latent ones, even though a marked variability in reactivity characterized patients with primary syphilis (Figure 1A). Mean absorbance shown for each peptide tested with each serum sample (Figure 2A) reiterated that patient sera recognized most prominently peptides 1, 4, and 5. In the Tp0435 experimentally determined protein structure [26,33], these peptides correspond to the region encompassing the α-helix preceding the soluble β-barrel structure (peptide 1), and the Tp0435 loop 1 (peptides 4 and 5), respectively (Figure 2A,B). Overall, a low degree of reactivity was detected to loops 2, 3, and 7 compared to loop 1, while peptides corresponding to loops 4–6 were virtually non-reactive (Figure 2B). Of the 63 sera tested, 93% (59/63) recognized peptide 4, 86% (54/63) recognized peptide 2, and 76% (48/63) recognized peptide 5. All secondary syphilis sera recognized all three peptides (Table 3). Of the four sera that failed to recognize peptide 4, two (one early latent serum and one late latent serum) were reactive to peptide 5, while two (one primary serum and one late latent serum) were reactive to peptide 1.

When reactivity was evaluated using syphilis history and HIV status as covariates, regardless of stage, peptides 1, 4, and 5 were generally recognized by a higher percentage of sera from patients with a history of syphilis infection, supporting that multiple exposures to the pathogen and to the abundant Tp0435 antigen facilitate recognition. HIV status, on the contrary, did not appear to affect recognition of the peptides alone or in combination. Regarding HIV status, however, all the sera used here were from patients in antiretroviral therapy.

### 3.2. Reactivity of Rabbit Sera to Tp0435 Peptides

**Figure 3:**
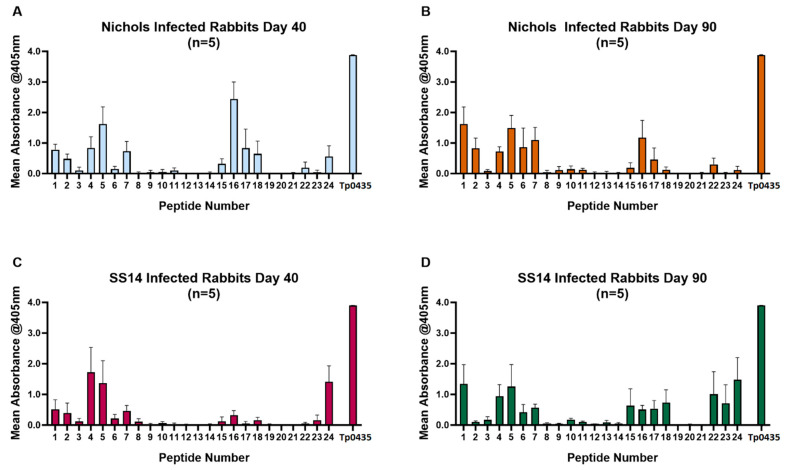
Reactivity of sera from experimentally infected rabbits to Tp0435 peptides. Mean reactivity to Tp0435 peptides in sera collected at day 40 post-infection (**A**) and day 90 post-infection (**B**) from rabbits inoculated with the Nichols strain of *T. pallidum*, and mean reactivity to Tp0435 peptides in sera collected at day 40 post-infection (**C**) and day 90 post-infection (**D**) from rabbits inoculated with the SS14 strain of *T. pallidum.* In all graphs, the Tp0435 label represents reactivity to wells where the full-length Tp0435 antigen was plated.

**Figure 4:**
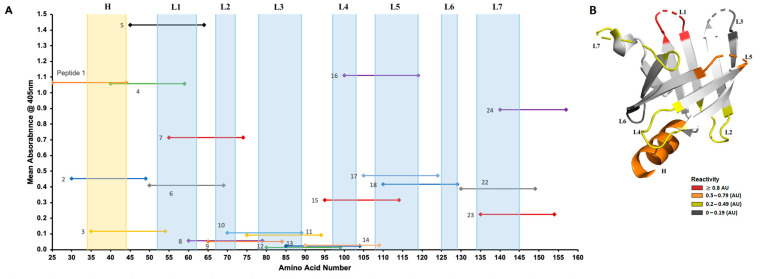
Mean reactivity of rabbit sera to individual peptides mapped to the Tp0435 protein structure. (**A**) Mean reactivity to individual peptides in rabbit sera independently of infecting *T. pallidum* strain. The *x* axis represents individual Tp0435 amino acids (25–159) without the cleavable signal peptide. Amino acids corresponding to the Tp0435 amino-terminal helix and loops are represented in the background highlighted in blue. (**B**) Tp0435 amino-terminal sequence and loops in rabbit sera highlighted in the experimentally determined protein structure based on mean reactivity in rabbit sera. Loops with a mean reactivity between ≤0.19 absorbance units (AUs) are highlighted in dark gray. Loops with a mean reactivity between 0.2 and 0.49 AUs are yellow, loops with a mean reactivity between 0.5 and 0.79 AUs are in orange, and loops with a mean reactivity ≥ 0.8 AUs are in red.

Rabbit sera collected from animals infected with the Nichols (*n* = 5) or SS14 (*n* = 5) strains overall showed a pattern of reactivity to peptides 1–9 (Figure 3A–D), not dissimilar to reactivity shown by human sera. In addition to a strong reactivity to peptides 1, 4, and 5, however, peptides 6 and 7 were more intensely recognized compared to patient sera, supporting that the amino-terminal helix and loop 1 are also targets of the humoral response in the rabbit model of infection. Overall, however, these sera also recognized peptides 15–18, predominantly encompassing Tp0435 loop 5. In the Tp0435 carboxyl terminus, reactivity to peptides 22–24 was prominent compared to peptides 19–21, representing upstream sequences. When sera collected at day 40 and day 90 were compared, reactivity to single peptides in general appeared to be similar or more elevated in day-90 serum specimens compared to day 40 (Figure 3A,B). A similar reactivity pattern was seen from sera collected at day 40 and day 90 post-infection from SS14-infected rabbits (Figure 3C,D). For each peptide tested with each rabbit serum, the mean absorbance (Figure 4A) confirmed that peptides 1, 4, 5, 16, and 24 were more intensely recognized. In the Tp0435 protein structure, these peptides correspond to the region preceding the β-barrel, loop 1, and loop 5 (Figure 4A,B). Overall, a higher degree of reactivity was seen for loop 2 and loop 7 compared to loop 4 and loop 6, while peptides corresponding to loop 3 were virtually non-reactive (Figure 4B).

## 4. Discussion

The *T. pallidum* Tp0435 antigen is a 14 kDa periplasmic lipoprotein encoded by one of the most highly expressed *T. pallidum* genes [35,36] and, consistently, one of the most abundant *T. pallidum* proteins, encompassing ~2% of the detergent-soluble protein content of this pathogen [37]. Furthermore, Tp0435 is the target of both an immunodominant humoral response and of the cellular immunity during experimental and/or natural syphilis [38,39,40]. There is an extensive body of evidence that *T. pallidum* Tp0435 and, in general, spirochetal lipoproteins are potent pro-inflammatory agonists and activators of the innate host response by binding to Toll-like receptors 2 and 1 on macrophages, monocytes, and dendritic cells to induce cellular activation [41,42,43,44,45], which explains their nature as immunodominant antigens. Given the above evidence, it is not surprising that TP0435 is in widespread use, either as the sole antigen or in combination with other *T. pallidum* lipoproteins, in treponemal tests that use recombinant proteins. Examples include the Roche Elecsys Syphilis, the Siemens Immulite 2000 Syphilis Screen, the Diasorin LIAISON Treponema Assay, the Fujirebio Lumipulse G TP-N assay, and the Zeus Scientific *T. pallidum* IgG Test System [46]. Detection of reactivity to Tp0435 is also a staple of Western blot-based tests, whether the assay uses a whole *T. pallidum* cell lysate or recombinant antigens. Overall, our work suggests that the use of Tp0435 as a diagnostic antigen could be limited to virtually three (peptides 1, 4, and 5) of the 24 peptides tested. Although most samples recognized peptide 4, the four sera that did not were able to recognize either one of the two other peptides, supporting that collective reactivity to these peptides could have allowed a diagnosis of infection. Although peptide 1 contains a truly specific sequence for *T. pallidum* and the human pathogens belonging to the *T. pallidum* subspecies *endemicum* and *pertenue*, peptides 4 and 5 include, respectively, the partial or complete GTLPAADCPGI sequence, which is fully conserved in the NlpE lipoproteins of *Prevotella/Segatella* bacteria species and of the opportunistic pathogen *Vibrio metoecus*. It is unclear, however, whether the contribution to the reactivity to peptides 4 and 5 that we observed in this study can be attributed to a response directed at the NlpE lipoproteins of these bacterial species, particularly from the commensal *Prevotella* species. However, this scenario seems unlikely, as the data we collected using sera from 40 syphilis-naïve patients did not show reactivity to the recombinant Tp0435 antigen used in this study. This supports the idea that reactivity to the GTLPAADCPGI epitope could specifically arise following infection with *T. pallidum*, although more data is needed to avail this hypothesis.

Despite being a soluble protein, immunity to Tp0435 is mostly directed to amino acid sequences external to the β-barrel scaffolding. Aside from the strongly recognized α-helix preceding the scaffolding, Tp0435-reactive epitopes fall onto the loops held by the protein β-strands (Figure 3 and Figure 4). This finding is not dissimilar from B-cell epitope mapping results for putative integral outer membrane proteins (OMPs) of *T. pallidum* [32,34,47]. Although the Tp0435 β-barrel is soluble, and the amphiphilic β-strands of bona fide OMPs are embedded in the outer membrane of the spirochete, it appears that amino acids composing the β-strands are less likely to be processed by antigen-presenting cells compared to the loop sequences, regardless of their location in *T. pallidum* ultrastructure.

It is also worth mentioning that, despite being a very abundant *T. pallidum* antigen, which per se supports a key role in syphilis pathogenesis and *T. pallidum* biology, the function of Tp0435 remains unclear. Initial clues about Tp0435’s function were provided by the crystal structure of the recombinant protein elucidated by Brautigam et al. [26,33]. The presence of a shallow basin-like depression formed by barrel residues led to the speculation that Tp0435 could be a receptor for a small ligand of unknown nature. Given that *T. pallidum* is metabolically crippled due to a systematic genomic reduction process that led it to become an obligate human pathogen with a strong dependence on the host for nutrient acquisition, one could further hypothesize that the hypothetical Tp0435 ligand would be essential for *T. pallidum* viability.

Although most of Tp0435 is undoubtedly localized in the periplasm of the syphilis agent, studies conducted recently by Chan et al. [48] based on the analysis of Tp0435 expressed in *Borrelia burgdorferi* (*B. burgdorferi*) and on electron microscopy on *T. pallidum* cells suggested that this protein could be processed into multiple isoforms with at least one variant scarcely and stochastically displayed on the spirochete surface, where it could work as an adhesin. Expression of Tp0435 in the poorly adherent *B. burgdorferi* B314 and B31HP strains allowed these strains to bind more efficiently to a variety of host cells. The ability of Tp0435 and other *T. pallidum* lipoproteins to gain access to the pathogen’s surface, however, is still a topic of debate in the field and requires further investigation centered on the syphilis pathogen, rather than a heterologous expression system, to produce more reliable data. Although there are no reports of immunization/challenge experiments using recombinant Tp0435, immunity to Tp0435 induced by injecting rabbits with Tp0435-expressing *B. burgdorferi* (used as a surrogate system) was not found to be protective to challenge with infectious *T. pallidum* in the rabbit model [49], which should be expected in the case of the surface localization of this antigen.

Lastly, our work showed several similarities but also differences when the analysis of human sera was compared to that of rabbit sera, with several peptides more strongly recognized in the animal model than in naturally infected individuals. These differences are likely due to the *T. pallidum* infectious dose that animals receive to induce immunity to the pathogen in a time frame compatible with performing experiments. Although the similarities in reactivity (e.g., to peptides 1, 4, and 5) suggest immunodominance for the epitopes harbored in these peptides, the differences (e.g., for peptides 6 and 7) highlight how results in the animal model alone cannot substitute for the analysis of clinical samples.

Limitations of this work include the relatively modest number of samples once the covariates (stage, syphilis stage, and HIV status) are factored in to draw definitive conclusions, even though a series of highly immunogenic peptides emerge even from using these samples. Another limitation could be considered the fact that the overlapping peptides (20 mers overlapping by 15 residues) will offer only limited resolution to pinpoint a specific epitope down to the 10-residue length. Future studies might utilize a higher number of peptides overlapping by fewer residues to increase the overall resolution and a more precise identification of the reactive epitope.

In conclusion, this study elucidates the immunodominant epitopes of the abundant *T. pallidum* Tp0435 protein, which could have translational applications in the development of diagnostic tests for this serious infection.

## Figures and Tables

**Table 1:** Characteristics of the participants enrolled in this study.

Stage (Number of Patients)	Provided Gender (Number)	Age Range (Median Age)	HIV Status (Number)	History of Syphilis Infection (Number)
Primary (22)	Man (20) Trans-woman (1) Woman (1)	20–46 (27.5)	Positive (11) Negative (11)	Positive (4) Negative (4) Unknown (14)
Secondary (7)	Man (7)	19–46 (38)	Positive (4) Negative (3)	Positive (3) Negative (1) Unknown (3)
Early Latent (22)	Man (19) Trans-woman (2) Woman (1)	18–37 (28.5)	Positive (5) Negative (17)	Positive (11) Negative (11)
Late Latent (12)	Man (10) Trans-woman (2)	25–54 (34.5)	Positive (6) Negative (6)	Positive (12)

**Table 2:** Peptides (20 mers overlapping by 15 amino acids), spanning the length of the Tp0435 mature antigen of *T. pallidum*.

Peptide # (aa Position) ^1^	Peptide Sequence ^2^
1 (25–44)	CTTVCPHAGKAKAEKVECAL
2 (30–49)	PHAGKAKAEKVECALKGGIF
3 (35–54)	AKAEKVECALKGGIFRGTLP
4 (40–59)	VECALKGGIFRGTLPAADCP
5 (45–64)	KGGIFRGTLPAADCPGIDTT
6 (50–69)	RGTLPAADCPGIDTTVTFNA
7 (55–74)	AADCPGIDTTVTFNADGTAQ
8 (60–79)	GIDTTVTFNADGTAQKVELA
9 (65–84)	VTFNADGTAQKVELALEKKS
10 (70–89)	DGTAQKVELALEKKSAPSPL
11 (75–94)	KVELALEKKSAPSPLTYRGT
12 (80–99)	LEKKSAPSPLTYRGTWMVRE
13 (85–104)	APSPLTYRGTWMVREDGIVE
14 (90–109)	TYRGTWMVREDGIVELSLVS
15 (95–114)	WMVREDGIVELSLVSSEQSK
16 (100–119)	DGIVELSLVSSEQSKAPHEK
17 (105–124)	LSLVSSEQSKAPHEKELYEL
18 (110–129)	SEQSKAPHEKELYELIDSNS
19 (115–134)	APHEKELYELIDSNSVRYMG
20 (120–139)	ELYELIDSNSVRYMGAPGAG
21 (125–144)	IDSNSVRYMGAPGAGKPSKE
22 (130–149)	VRYMGAPGAGKPSKEMAPFY
23 (135–154)	APGAGKPSKEMAPFYVLKKT
24 (140–159)	KPSKEMAPFYVLKKTKK

^1^ Amino acids 1–24 encompass the cleavable signal peptide and are not in the mature protein. ^2^ Peptides associated to loops based on the Tp0435 experimentally determined structure [33] are underlined. L1: TLPAADCPGID; L2: FNADGT; L3: LALEKKSAPSPL; L4: VREDGIV; L5: VSSEQSKAPHEK; L6: IDSNS; L7: GAPGAGKPSKEM. The amino-terminal α-helix amino acids (KAKAEKVECALK) are boxed.

**Table 3:** Serological reactivity to individual peptides by disease stage, history of syphilis, and HIV status.

Antigen	All Sera (63)	Primary Syphilis Sera (22)	Secondary Syphilis Sera (7)	Early Latent Syphilis Sera (22)	Late Latent Syphilis Sera (12)	Syphilis History ^1^	HIV Status ^2^
Peptide 1	86% (54/63)	86% (19/22)	100% (7/7)	82% (18/22)	83% (10/12)	Y: 44% N: 30% U: 26%	P: 44% N: 54%
Peptide 4	94% (59/63)	95% (21/22)	100% (7/7)	95% (21/22)	83% (10/12)	Y: 42% N: 29% U: 29%	P: 48% N: 52%
Peptide 5	76% (48/63)	77% (17/22)	100% (7/7)	73% (16/22)	67% (8/12)	Y: 44% N: 28% U: 28%	P: 49% N: 51%
Peptide 1, 4, and 5	68% (43/63)	68% (15/22)	100% (7/7)	63% (14/22)	58% (7/12)	Y: 42% N: 28% U: 30%	P: 44% N: 56%
Peptide 1, 4, or 5	100% (63/63)	100% (22/22)	100% (7/7)	100% (22/22)	100% (12/12)	Y: 45% N: 42% U: 13%	P: 42% N: 58%

^1^ Regardless of stage. Y: with documented history of infection; N: without documented history of infection, U: unknown. ^2^ Regardless of Stage. P: HIV positive; N: HIV negative.

## Data Availability

The original contributions presented in this study are included in the article. Further inquiries can be directed to the corresponding author.
